# Managing Acute STEMI in the Setting of Immune Thrombocytopenic Purpura: A Therapeutic Dilemma

**DOI:** 10.1016/j.jaccas.2026.109902

**Published:** 2026-08-19

**Authors:** Khaleed Jemmieh, Mohamed Tawengi, Jawaher Baraka, Fathima Mahmood, Fatima Alzahraa Alhaj Akeel, Abdelkarim Alammora, Mhd Ghith Choura

**Affiliations:** a Cardiology Department, Hamad Medical Corporation, Doha, Qatar; b Internal Medicine Department, Hamad Medical Corporation, Doha, Qatar; c Medical Internship Department, Hamad Medical Corporation, Doha, Qatar

**Keywords:** antiplatelet, myocardial infarction, thrombosis

## Abstract

**Background:**

Immune thrombocytopenia (ITP) is an autoimmune condition characterized by thrombocytopenia. Despite bleeding tendencies, patients with ITP face thrombotic risks. Acute coronary syndrome in ITP is uncommon and poses a management dilemma between antithrombotic therapy and the high bleeding risk.

**Case summary:**

A 43-year-old man with severe ITP (platelet nadir 1 × 10^3^/μL) treated with avatrombopag presented with inferoposterior ST-elevation myocardial infarction (STEMI). Angiography showed occlusion of the right posterolateral ventricular branch and right posterior descending artery. Plain old balloon angioplasty was performed without stenting. He remained stable without bleeding and was discharged on dual antiplatelet therapy, avatrombopag, and prednisolone.

**Discussion:**

ITP presents a clinical paradox of concurrent bleeding and clotting risks. Managing STEMI requires balancing these competing mechanisms.

**Take-Home Messages:**

Thrombocytopenia in ITP does not protect against acute coronary syndrome. Navigating the balance between bleeding and clotting following a STEMI event requires a tailored, flexible approach.

## Visual Summary

**Figure:**
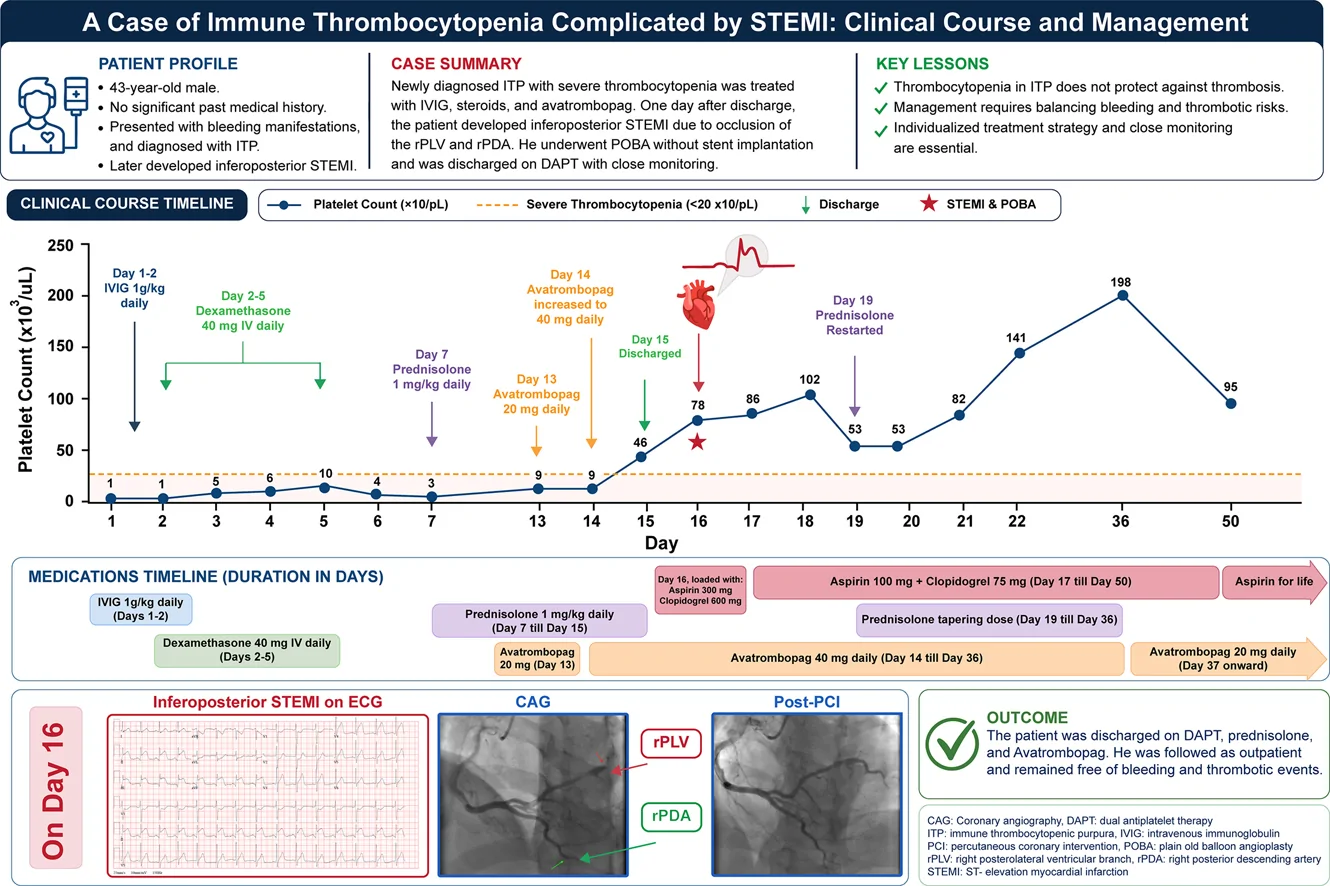

Immune thrombocytopenia (ITP) is an autoimmune disorder characterized by thrombocytopenia secondary to platelet destruction and decreased platelet production.^1^ Patients with ITP usually present with mucocutaneous bleeding, and in severe cases, they may develop significant hemorrhage. However, despite thrombocytopenia, patients still have a risk of thrombosis. Increasing evidence has shown that ITP is associated with a considerable risk of arterial and venous thromboembolism. This is believed to be multifactorial, including disease, patient, and treatment-related mechanisms.^2^^,^^3^

Acute coronary syndrome (ACS) in patients with ITP is uncommon and challenging to manage. Reperfusion and antithrombotic therapies are warranted but must be weighed against the risk of bleeding because of severe thrombocytopenia and unpredictable platelet count trends. Furthermore, ITP treatment, which typically includes corticosteroids, intravenous immunoglobulins, and thrombopoietin receptor agonists, may increase the risk of thrombosis.^4^ Evidence on management of ACS in patients with ITP is still limited, and a recent systematic review only identified 32 published cases.^4^ Here, we report a case of inferoposterior ST-segment elevation myocardial infarction (STEMI) occurring shortly after treatment escalation for newly diagnosed ITP, highlighting the risk of coronary thrombosis despite thrombocytopenia and the challenges of revascularization and antiplatelet management in such cases.

## History of Presentation

A 43-year-old man presented with a 10-day history of epistaxis, black stools, and fresh rectal bleeding. Physical examination revealed diffuse petechiae and purpura involving both upper and lower limbs and the trunk. He was diagnosed with ITP, hospitalized for 15 days, then discharged home following treatment and stabilization.

Twenty-four hours after discharge, the patient experienced severe chest pain, prompting emergency medical services and readmission. He was vitally stable and the physical examination was unremarkable.

## Past Medical History

No significant past medical history.

## Investigations

During the initial presentation, investigations revealed severe thrombocytopenia with a platelet count of 1 × 10^3^/μL, hemoglobin of 11.5 g/dL, and a normal white blood cell count. Subsequent work-up concluded that ITP was the most likely diagnosis. Stool antigen testing for *Helicobacter pylori* (*H pylori*) was positive. Platelet count upon discharge was 46 × 10^3^/μL.

During his second presentation for chest pain, his platelet count was 78 × 10^3^/μL. Electrocardiogram showed ST-segment elevation in leads II, III, and augmented vector foot, with reciprocal ST-segment depression in leads augmented vector left, V_1_, and V_2_, confirming an inferoposterior STEMI (Figure 1). Emergency coronary angiography revealed complete occlusion of the right posterolateral ventricular branch and right posterior descending artery (Figure 2).

**Figure 1:**
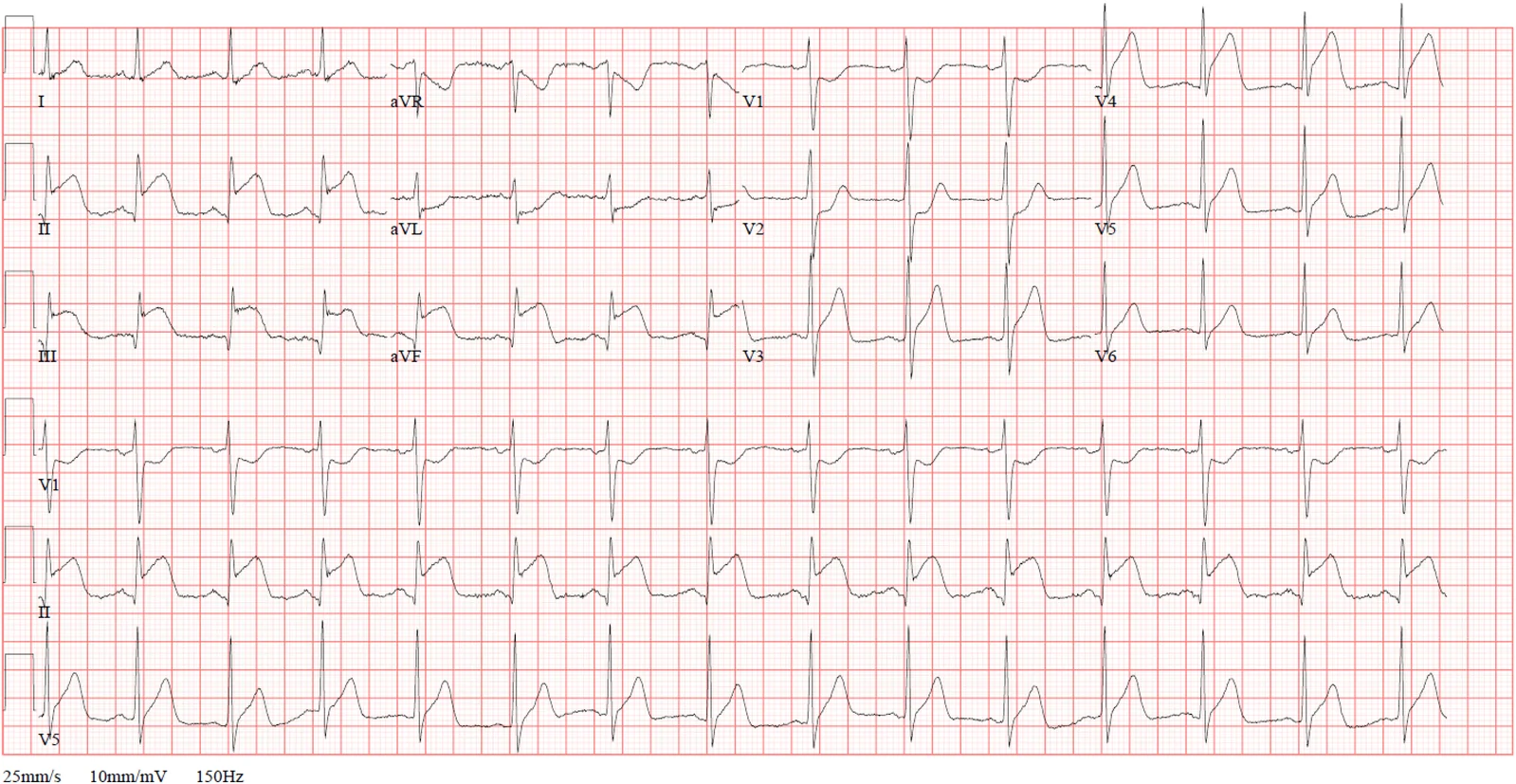
12-Lead Electrocardiogram Demonstrating Sinus Rhythm With ST-Segment Elevation in Leads II, III, and aVF, With Reciprocal ST-Segment Depression in Leads aVL, V_1_, and V_2_, Consistent With an Acute Inferoposterior ST-Segment Elevation Myocardial Infarction

**Figure 2:**
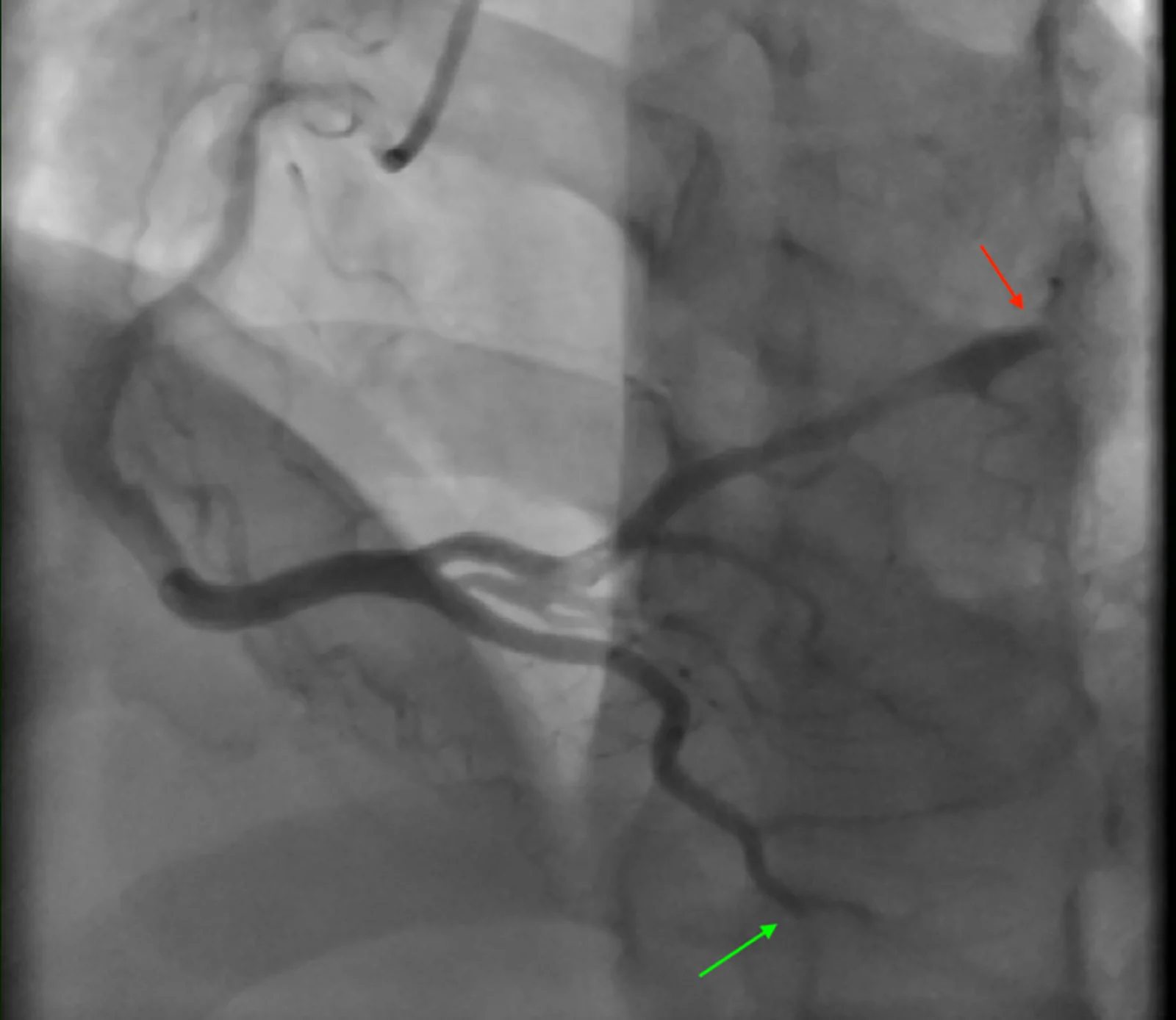
Coronary Angiogram of the Right Coronary System Showing 100% Stenosis in Right Posterolateral Ventricular Branch (Red Arrow) and Right Posterior Descending Artery (Green Arrow)

## Management

Phase 1 (Initial ITP Management): During the initial admission, the patient was transfused with one adult unit of platelets and started on medical therapy consisting of intravenous dexamethasone (40 mg daily) and intravenous immunoglobulin (1 g/kg daily). Quadruple eradication therapy was initiated for the *H pylori* infection. Because of persistent epistaxis, hematuria, and a platelet count below 10 × 10^3^/μL for 13 days, oral avatrombopag was initiated at 20 mg daily and uptitrated to 40 mg daily the following day. He was discharged on oral prednisolone 1 mg/kg (60 mg), avatrombopag (40 mg daily), and quadruple therapy for *H pylori*.

Phase 2 (STEMI Management): Upon the second presentation with STEMI, aspirin 300 mg and clopidogrel 600 mg loading doses were administered. The patient underwent plain old balloon angioplasty (POBA) of both the right posterolateral ventricular branch and right posterior descending artery vessels (Figure 3), receiving 6,000 units of unfractionated heparin during the intervention. Coronary stent implantation was deliberately avoided to minimize the mandatory duration and intensity of subsequent antiplatelet therapy. Following the intervention, prednisolone was temporarily held on admission while avatrombopag was maintained. When his platelet count dropped acutely from 102 to 53 × 10^3^/μL on day 3 post-STEMI, prednisolone was resumed.

**Figure 3:**
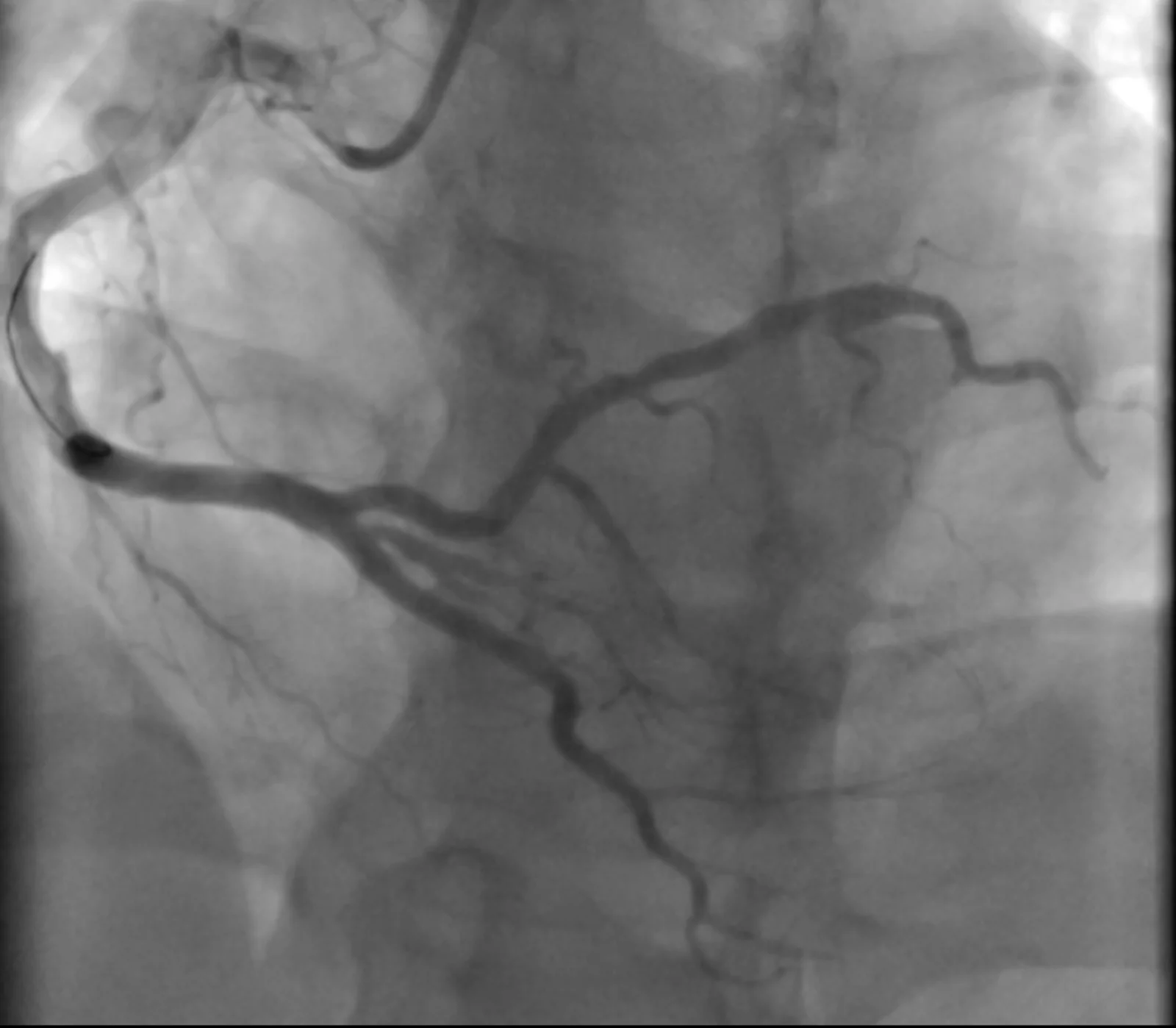
Coronary Angiogram of the Right Coronary System After Plain Balloon Angioplasty on Both Right Posterolateral Ventricular Branch and Right Posterior Descending Artery A good result is noted with TIMI flow grade 3 achieved.

## Outcome and Follow-Up

Following the POBA intervention, the patient remained stable and symptom-free. On day 6 post-STEMI, his platelet count reached 141 × 10^3^/μL, and he was subsequently discharged home in stable condition.

His discharge regimen included avatrombopag 40 mg daily and a tapering dose of prednisolone 60 mg daily for ITP. For STEMI, he was prescribed dual antiplatelet therapy (DAPT), with aspirin 100 mg daily and clopidogrel 75 mg daily for 1 month, to be followed by aspirin monotherapy for life.

At 2 weeks postdischarge, the patient's platelet count was 198 × 10^3^/μL, and the hematology team decreased the avatrombopag maintenance dose to 20 mg daily. At his 1-month follow-up, his platelet count was 95 × 10^3^/μL. Clinically, the patient tolerated his first month of DAPT exceptionally well with no evidence of bleeding complications or recurrent thrombotic events.

A graphical abstract including a summary of the case and key events is shown in the Visual Summary.

## Discussion

ITP is considered a bleeding disorder; however, evolving case reports have illustrated that patients still could develop thrombotic events. In this case, despite the high bleeding risk initially and after initiation of ITP treatment, the patient developed acute STEMI. Studies have demonstrated that patients with ITP are more susceptible to developing both arterial and venous thrombosis compared with those without ITP.^2^ However, establishing a definitive causal relationship between ITP and thrombosis remains challenging. Understanding this paradox is crucial in helping clinicians identify the incidence, risks, and proper management strategies, as both bleeding and thrombosis may lead to catastrophic and life-threatening events, bleeding or thrombosis. A 2-sample Mendelian randomization study presents empirical support for the causal relationship between ITP and thrombosis, and it is important to note that a diminished platelet count does not serve as a preventive measure against thrombus formation.^5^ Many possible mechanisms have been proposed to support this association. The platelets in patients with ITP are occasionally immature and undergo some changes in response to the disease process that make these platelets pro-thrombotic. These platelet particles are larger, more adhesive, and release more platelet-derived microparticles compared with those in individuals without ITP.^6^^,^^7^ Secondly, ITP treatment plays a role in increasing the thrombotic risk. In this case, it was evident from the drastic response to therapy after receiving intravenous immunoglobulin, steroids, and avatrombopag. In fact, many case reports have reported thrombotic events following ITP treatment.^2^

The burden of this paradoxical combination is challenging to manage and predict, as therapy should aim to prevent both bleeding and thrombosis. In this case, the decision to proceed with POBA without stent implantation was made to decrease the duration of DAPT. The intended duration of DAPT was left flexible and subject to reassessment based on future platelet counts and bleeding risk, highlighting the need for an individualized treatment strategy rather than rigid adherence to a fixed treatment duration.

A large database study conducted in the US showed that patients with ITP and acute MI were less likely to undergo revascularization, had more bleeding complications, more bare-metal stent use, but had similar short-term in-hospital mortality.^7^ However, in another 15-year database study, 10% in-hospital mortality was reported, higher than overall ITP hospitalizations (4%), with transfusions and stent placement predicting death.^8^

Growing evidence of thrombotic complications in ITP underscores the need for a multidisciplinary approach to short- and long-term care. Collaboration between hematology and cardiology is vital, as therapeutic risks and benefits are deeply intertwined. Ultimately, clinicians must recognize that a low platelet count in ITP does not prevent myocardial infarction; rather, it can actively contribute to acute coronary syndromes via complex disease mechanisms and treatment-induced fluctuations.

## Conclusions

This case underscores that ITP does not confer protection against thrombotic events, including STEMI. Managing concurrent acute coronary syndrome and severe thrombocytopenia presents a clinical paradox that demands a dynamic balance between bleeding and thrombotic risks. Ultimately, an individualized antiplatelet strategy combined with multidisciplinary monitoring is essential to optimize ischemic outcomes and mitigate hemorrhagic complications.

**Take-Home Messages**

• Thrombocytopenia in immune thrombocytopenia does not protect against acute coronary syndrome, creating a clinical paradox of concurrent bleeding and thrombotic risks.
• Managing ST-segment elevation myocardial infarction in immune thrombocytopenia requires a tailored approach; balloon angioplasty without stenting minimizes the duration of dual antiplatelet therapy.

**Visual Summary:**
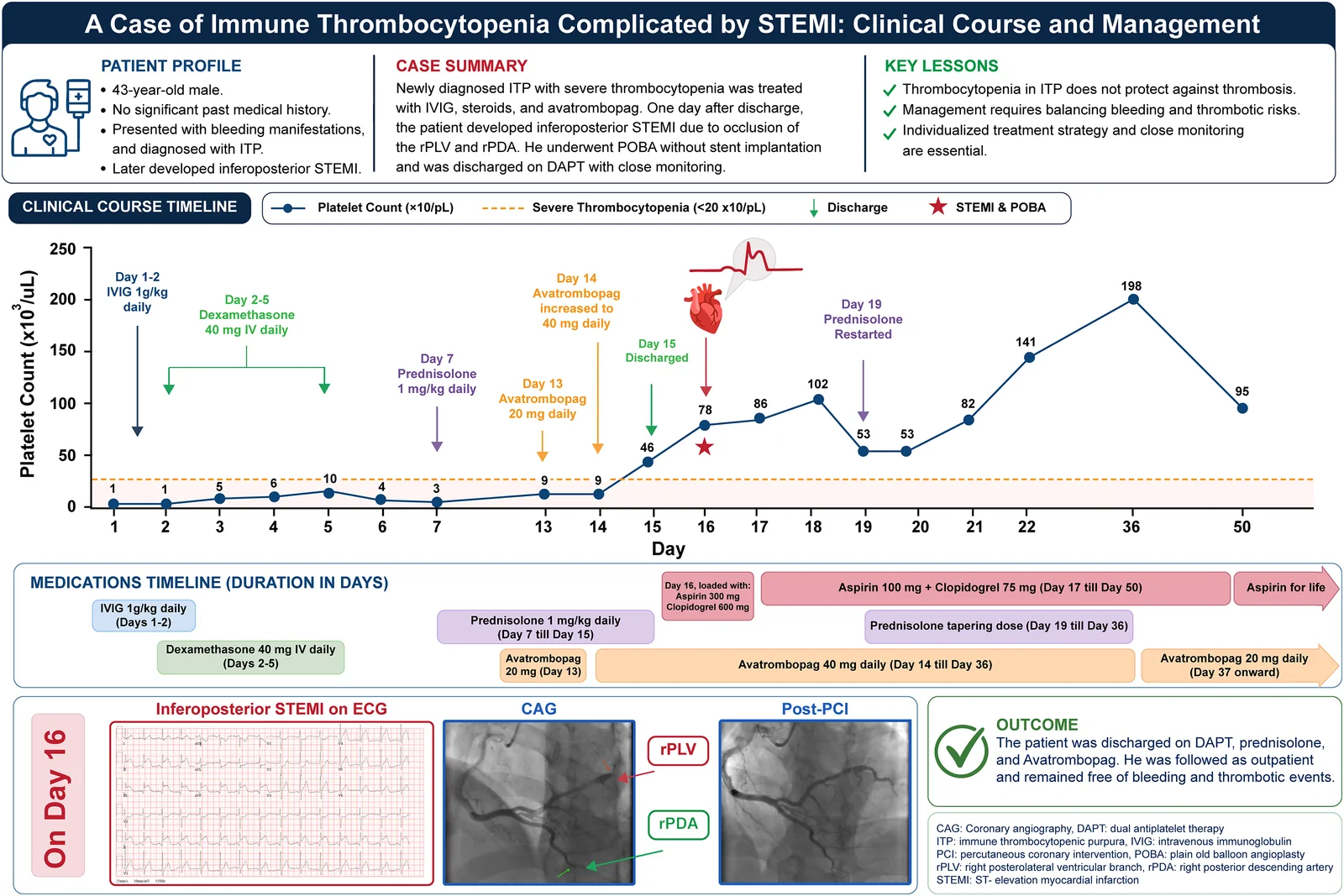
A Summary of the Case Including Patient Profile, Case Summary, Platelet Count Trend, Investigations, Treatment Timeline, Outcome, and Key Lessons CAG = coronary angiography; DAPT = dual antiplatelet therapy; ECG = electrocardiogram; ITP = immune thrombocytopenia; IV = intravenous; IVIG = intravenous immunoglobulin; PCI = percutaneous coronary intervention; POBA = plain old balloon angioplasty; rPDA = right posterior descending artery; rPLV = right posterolateral ventricular branch; STEMI = ST-segment elevation myocardial infarction.

## Funding Support and Author Disclosures

The authors have reported that they have no relationships relevant to the contents of this paper to disclose.
